# Catheter-related gas-forming suppurative thrombophlebitis

**DOI:** 10.1186/s12245-024-00602-8

**Published:** 2024-03-04

**Authors:** Yasuyoshi Miyamura, Takeshi Shimazaki, Kunihiko Okada

**Affiliations:** Department of Emergency and Critical Care Medicine, Saku Central Hospital Advanced Care Center, Nakagomi, Saku, Nagano 3400-28 Japan

**Keywords:** Catheter-related bloodstream infection, Central line-associated bloodstream infection, Central venous catheter-related thrombosis, *Clostridium perfringens*, Gas-forming suppurative thrombophlebitis

## Abstract

Catheter-related suppurative thrombophlebitis (CRST) is a complication of catheter-related bloodstream infection (CRBSI). The microbiology of CRST is similar with the microbiology of CRBSI, but *Clostridium perfringens* that causes gas gangrene is a rare pathogen of CRBSI and CRST. We present a case of catheter-related gas-forming suppurative thrombophlebitis due to *Clostridium perfringens* infection. Gas-forming thrombus around the catheter can be useful findings for the early diagnosis of catheter-related clostridial thrombophlebitis.

## Case presentation

**Fig. 1:**
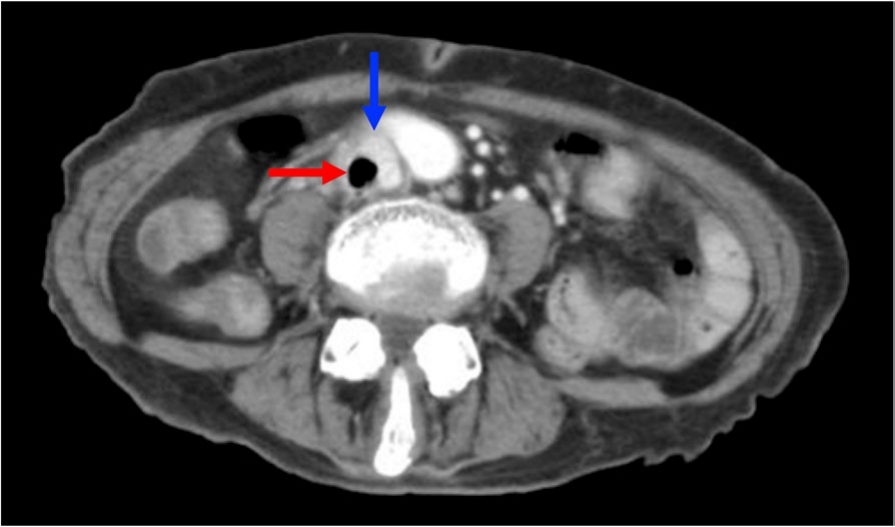
Axial contrast-enhanced computed tomography (CECT) showing round gas in a thrombus (red arrow) in the inferior vena cava (blue arrow)

**Fig. 2:**
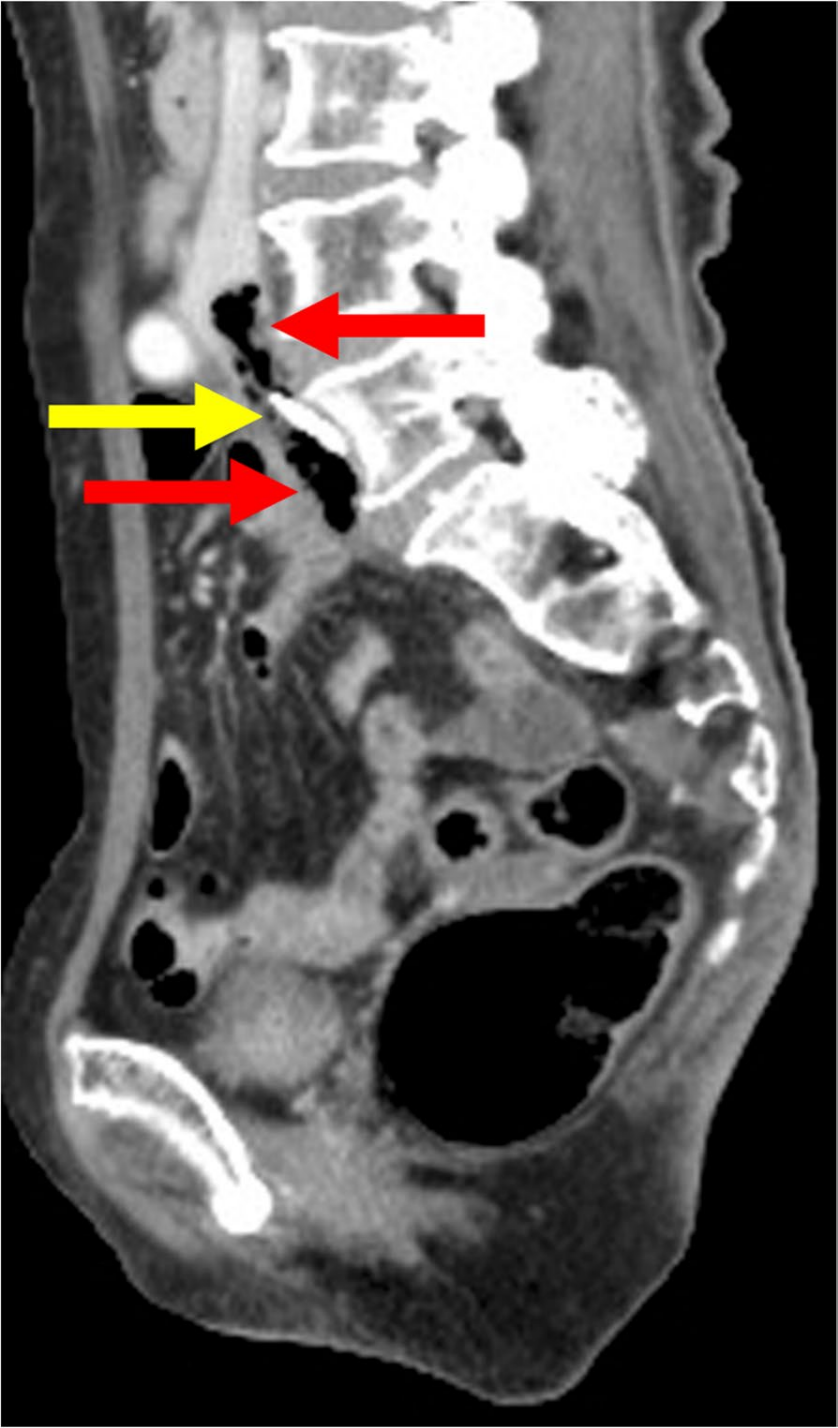
Sagittal CECT showing columnar gas (8.5 mm in diameter and 60 mm in length) in a thrombus (red arrows) and the central catheter tip in the right common iliac vein (yellow arrow)

A 78-year-old woman with diabetes mellitus was admitted to a hospital owing to a 3-month history of anorexia. Seven days before, a central venous catheter was inserted in the right femoral vein for total parenteral nutrition. She was referred to our hospital owing to a 2-day history of fever. Ultrasonography detected thrombus in the inferior vena cava. Further evaluation was performed by contrast-enhanced computed tomography (CECT). CECT revealed the presence of columnar gas in a thrombus around the catheter tip, extending from the right common iliac vein to the inferior vena cava (Figs. 1 and 2). Blood and removed catheter tip cultures were all positive for *Clostridium perfringens*. She received treatment with intravenous ampicillin-sulbactam for two weeks, followed by oral amoxicillin for two weeks. Anticoagulation therapy was continued for three months, and thrombus was dissolved. Recurrence was not observed following treatment.

## Diagnosis

### Catheter-related gas-forming suppurative thrombophlebitis due to *Clostridium perfringens* infection

The risk of catheter-related bloodstream infection (CRBSI) and catheter-related thrombosis was higher for femoral compared with subclavian and internal jugular [1]. Catheter-related suppurative thrombophlebitis (CRST) is a relatively uncommon complication of CRBSI [2], but should be ruled out [3]. The common pathogen is *Staphylococcus aureus* [2]. In the current case, *Clostridium perfringens* probably invaded the percutaneous tract or was carried hematogenously to the catheter because of bacterial translocation. Therefore, thrombus formation around the catheter was infected with *Clostridium perfringens*, resulting in quite rare form of central line-associated bloodstream infection, gas-forming suppurative thrombophlebitis. Our case highlights that gas-forming thrombus around the catheter can be useful findings that early diagnose catheter-related clostridial thrombophlebitis.

## Data Availability

No datasets were generated or analysed during the current study.

## Abbreviations

CRST: Catheter-related suppurative thrombophlebitis
CRBSI: Catheter-related bloodstream infection
CECT: Contrast-enhanced computed tomography
